# The complete plastome of *Nannoglottis ravida*, an extremely endangered species in the Qinghai-Tibet Plateau

**DOI:** 10.1080/23802359.2020.1834887

**Published:** 2020-11-13

**Authors:** Bibo Yang, Tianqi He, Lushui Zhang

**Affiliations:** Key Laboratory of Bio-Resource and Eco-Environment of Ministry of Education, College of Life Sciences, Sichuan University, Chengdu, China

**Keywords:** *Nannoglottis ravida*, plastome, endangered species, phylogenetic relationship

## Abstract

*Nannoglottis ravida* is an extremely endangered species in the Qinghai-Tibet Plateau. Based on the second-generation high-throughput genome sequencing, we assembled the plastome of this species. The length of the total plastome is 152,324 bp with a typical quadripartite structure including a large single-copy region of 83,708 bp, a small single-copy region of 29,882 bp and two reverse repeat regions of 19,367 bp respectively. A total of 131 genes were annotated including 85 protein-coding genes (PCG), 36 tRNA genes, 8 rRNA genes and 2 pseudogenes. The constructed phylogenetic tree with other species of two tribes Senecioneae and Astereae based on plastomes suggests that *N. ravida* has a close relationship with the Astereae, but diverged early from this tribe.

*Nannoglottis ravida* Maximowicz (Asteraceae) is an extremely endangered species in the eastern Qinghai-Tibet Plateau. Only two to three populations fewer than 500 individuals were recorded in the dry and stony valleys at altitudes between 3700 and 4100 m (Liu et al. 2002; Gao 2004). This species is an alpine subshrub with woody roots and trimorphous flowers. It was firstly placed in the genus *Senecio* and later transferred into *Nannoglottis* (Jeffrey and Chen 1984). Morphological, karyotypic and molecular evidence supported such a taxonomic treatment (Liu et al. 2000, 2002; Zhang 2000). However, this species diverged very early from the other seven species of the genus when the plateau uplifted 3.4 million years ago (Liu et al. 2002). It is better to treat it as a monotypic section because of the extremely genetic and morphological divergence (Gao and Chen 2005). In addition, phylogenetic relationship of the re-circumscribed genus *Nannoglottis* remains disputed although it is more reasonable to place it in the tribe Astereae with an isolated position than in the Senecioneae (Liu et al. 2002). Chloroplast genomes (Plastomes) were reported for numerous endangered species (e.g. Tanja et al. 2018; Yang et al. 2018; Zhang et al. 2018). However, little is known on the plastome information of *N. ravida.* Herein, we reported the plastome of *N. ravida*. The annotated plastome has been submitted to National Center for Biotechnology Information (NCBI) database with an accession number MT767106 (https://www.ncbi.nlm.nih.gov/nuccore/MT767106).

We collected fresh leaves of wild *N. ravida* from Chengduo in Qinhai Province, China (96°59′8″E, 33°21′24″N) and dried and stored them in the plastic bags using silica gels. Voucher specimen of this species was reserved in the Key Laboratory of Bio-resource and Eco-environment of Ministry of Education (Sichuan, China). A modified CTAB method (Doyle and Doyle 1987) was used to extract the total DNAs of the dried leaves of this species. The paired-end libraries with insert size of 500 base pairs (bp) was constructed and HiSeq X Ten System were used to sequence these libraries. We used 6 Gb raw read data to be filtered with the fast QC (Gdula et al. 2019) and Trimmomatic (Bolger et al. 2014). We downloaded the plastome of *Lagenophora cuchumatanica* (GenBank accession number NC034819) as the reference and assembled the clean data into the plastome of *N. ravida* using NOVOplasty v4.1 (Dierckxsens et al. 2016). BWA v.0.7.12 (Li and Durbin 2009) and SAMtools v.1.2（Li et al. 2009）were used to connect and compare the referenced and the assembled sequences. We also used Geneious v.R.8.1.4（Kearse et al. 2012）to adjust the targeted sequence manually. We used the Plann and Sequin (NCBI website) (Huang and Cronk 2015) to annotate the newly obtained plastome.

The plastome of *N. ravida* is 152,324 bp in length with a typical quadripartite structure, which comprises a large single-copy region (LSC) of 83,708 bp, a small single-copy region (SSC) of 29,882 bp and two reverse repeat regions (IRA and IRB) of 19,367 bp each. The overall GC content of the plastome is 37.42% and the LSC, SSC, and IRs regions occupy 35.53%, 38.02%, and 41.04% respectively. Plastome annotation revealed a total of 131 genes, containing 85 protein-coding genes (PCG), 36 transfer RNA (tRNA) genes, 8 ribosomal RNA (rRNA) genes, and 2 pseudogenes. Most of these genes are single copy genes, while there are 15 genes (6 PCGs, 7 tRNA genes, 4 rRNA genes) were duplicated in the IR regions. Both *rps19* and *ycf1* genes in the IRb and SSC regions of the available plastomes in the other Astereae species were annotated as the pseudogenes in *N. ravida*.

In order to construct phylogenetic relationship of this species and other species of the tribes Senecioneae and Asteraceae, we downloaded 11 plastome sequences (the GenBank accession numbers of each species in Figure 1) of these two tribes with *Praxelis clematidea* of another tribe as outgroup. We extracted all coding sequences from each plastome by *Python Script* and comprised them as a dataset for phylogenetic analyses. These sequences were aligned using the software MAFFT (Katoh and Standley 2013). A Maximum Likelihood Analysis was performed by RAxML v8.2.9 (Stamatakis 2014) with GTRGAMMA set as the best model. We carried out 1000 bootstrap tests to examine statistical supports for each clade. The constructed phylogenetic tree suggested that *N. ravida* diverged from the common ancestor of the Astereae species early (Figure 1) but distantly related to the Senecioneae as suggested before (Liu et al. 2002).

**Figure 1.**
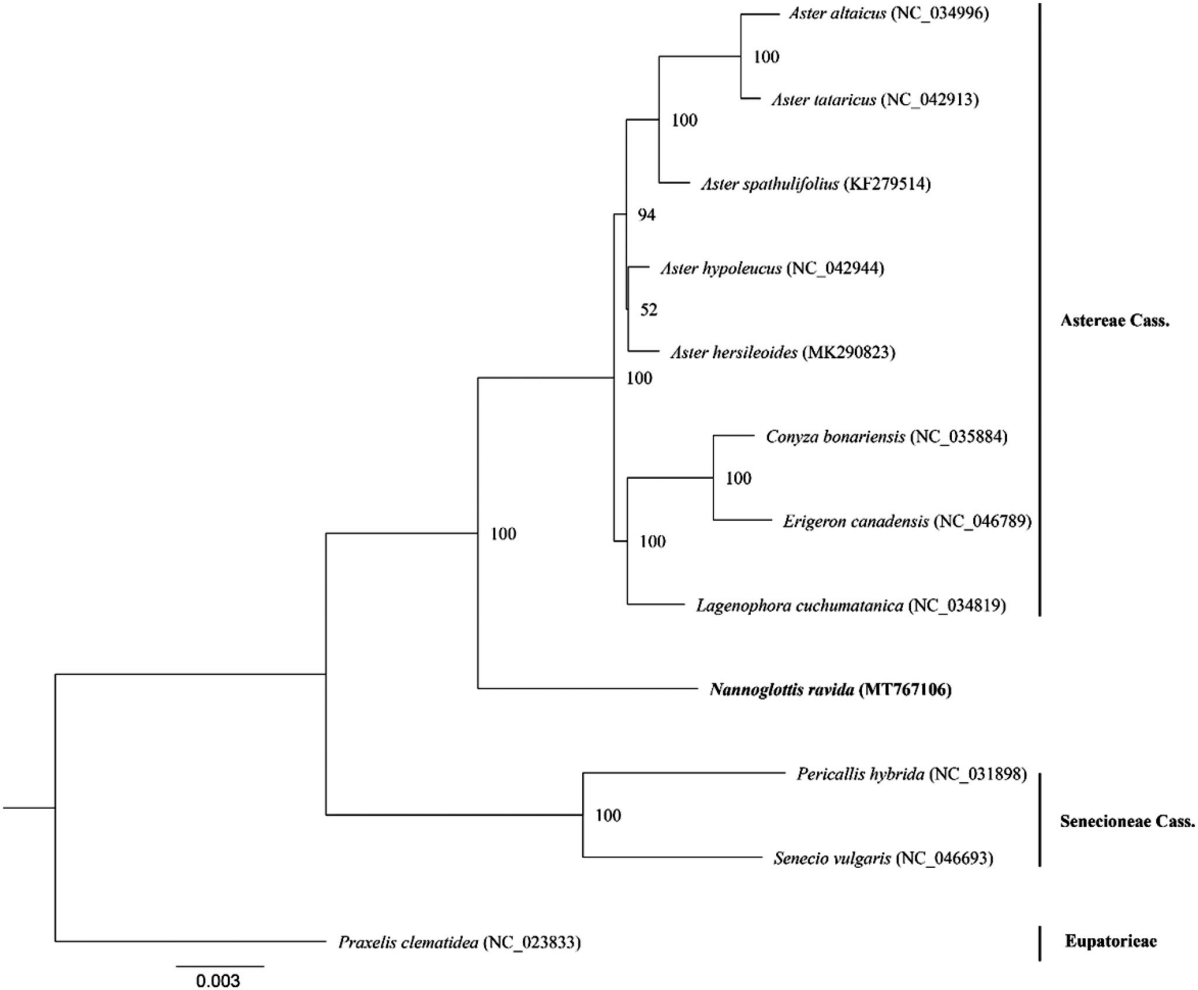
A phylogenetic tree based on the complete plastome sequences of *Nannoglottis ravida* and other 11 species of the tribes Asteraceae and Senecioneae. Numbers in the nodes are the bootstrap values from 1000 replicates, and the GenBank accession number for each plastome is listed in brackets, respectively.

The reported plastome here provides an important resource for designing plastome SSR markers to examine genetic diversity and gene flow through seeds in the endangered *N. ravida* because the plastomes of most angiosperms are maternally inherited (Zhang et al. 2005; Wang et al. 2009). In addition, with more platomes available for more *Nannoglottis* species in the future, the systematic position and diversification history of this genus can be further evaluated powerfully at the genomic level (Liu et al. 2002).

## Data Availability

The chloroplast genome sequence reported here for *Nannoglottis ravida* in the present paper has been submitted to National Center for Biotechnology Information (NCBI) database with an accession number MT767106, which is publicly accessible at https://www.ncbi.nlm.nih.gov/nuccore/MT767106
